# The persistent southern disadvantage in US early life mortality, 1965–2014

**DOI:** 10.4054/demres.2020.42.11

**Published:** 2020-02-25

**Authors:** Nathan T. Dollar, Iliya Gutin, Elizabeth M. Lawrence, David B. Braudt, Samuel H. Fishman, Richard G. Rogers, Robert A. Hummer

**Affiliations:** 1 University of North Carolina at Chapel Hill, USA; 2 University of North Carolina at Chapel Hill, USA; 3 University of Nevada, Las Vegas, USA; 4 Duke University, Durham, NC, USA; 5 University of Colorado Boulder, USA

## Abstract

**BACKGROUND:**

Recent studies of US adult mortality demonstrate a growing disadvantage among southern states. Few studies have examined long-term trends and geographic patterns in US early life (ages 1 to 24) mortality, ages at which key risk factors and causes of death are quite different than among adults.

**OBJECTIVE:**

This article examines trends and variations in early life mortality rates across US states and census divisions. We assess whether those variations have changed over a 50-year time period and which causes of death contribute to contemporary geographic disparities.

**METHODS:**

We calculate all-cause and cause-specific death rates using death certificate data from the Multiple Cause of Death files, combining public-use files from 1965–2004 and restricted data with state geographic identifiers from 2005–2014. State population (denominator) data come from US decennial censuses or intercensal estimates.

**RESULTS:**

Results demonstrate a persistent mortality disadvantage for young people (ages 1 to 24) living in southern states over the last 50 years, particularly those located in the East South Central and West South Central divisions. Motor vehicle accidents and homicide by firearm account for most of the contemporary southern disadvantage in US early life mortality.

**CONTRIBUTION:**

Our results illustrate that US children and youth living in the southern United States have long suffered from higher levels of mortality than children and youth living in other parts of the country. Our findings also suggest the contemporary southern disadvantage in US early life mortality could potentially be reduced with state-level policies designed to prevent deaths involving motor vehicles and firearms.

## 1. Introduction

Several recent studies demonstrate that mortality rates and life expectancy in the United States vary extensively by county, state, and region of the country, and that these disparities have increased in recent decades (Cossman et al. 2010; Dwyer-Lindgren et al. 2017; Fenelon 2013; Montez and Berkman 2014; Sparks and Sparks 2010). At the state and regional levels, widely cited studies have documented an increasing mortality disadvantage in southern states and in the region defined as Appalachia (Fenelon 2013; Mokdad et al. 2018; Montez and Berkman 2014; Singh 2010; Singh, Kogan, and Slifkin 2017). Most of this work focuses on adult mortality. In this article, we contribute to the literature by examining variations in early life (ages 1 to 24)^6^ mortality rates at the state and census division levels. We examine whether that variation has changed over a 50-year time period and identify which causes of death contribute to contemporary geographic disparities.

Most studies of geographic trends and patterns in US mortality focus on rates among adults, which is expected, given that the vast majority of deaths occur after the age of 50. Indeed, eight of the ten leading causes of death in the United States are related to chronic and degenerative diseases that predominate in old age (Heron 2017). Although US death rates among children, youth, and adolescents are much lower compared to adults, early life mortality rates in the United States are much higher than in high-income peer countries (Cunningham, Walton, and Carter 2018; National Research Council 2013). With a few notable exceptions (see Singh 2010; Singh et al. 2013), very little work focuses on trends and geographic patterning of early life mortality in the United States. This is an unfortunate gap given that early life deaths contribute disproportionately to years of life lost and have devastating effects on families and communities. Moreover, most deaths in early life are from external causes and thus potentially amenable to policy interventions. Although individual health behaviors – primarily smoking and diet – explain much of the southern disadvantage in US mortality among middle-aged and older adults (Fenelon 2013; Tencza, Stokes, and Preston 2014), such behaviors are unlikely to have major impacts on patterns and trends in early life mortality. Recent research also shows that international disparities in overall life expectancy – such as that between the United States and its high-income peers (Ho 2013) – are strongly influenced by high US early life mortality, particularly due to external causes. The extent to which geographic disparities in mortality are observed within the United States warrants further investigation, especially in early life.

In this article, we seek to fill the gap in our understanding of the patterns and trends in early life mortality in the United States. We build on past research documenting diverging geographic trends in US adult mortality; this work finds that the southern states and census divisions have increasingly fallen behind the rest of the nation and now exhibit the highest adult mortality rates (Fenelon 2013). Our study focuses on two research questions specific to US early life mortality. First, over the past 50 years, how has the geographic patterning of US early life mortality at the state and census division levels changed? Second, which causes of death are most responsible for contemporary geographic disparities in early life mortality? Although there is no consensus regarding which levels of analysis are optimal for examining geographic disparities in population health, we focus our analysis on states and census divisions. We focus on states because we suspect that many deaths occurring in early life can be reduced through state-level policy interventions such as those regulating traffic safety and firearms. We aggregate to the census division because evidence suggests the most disadvantaged states tend to cluster in the East South Central (Alabama, Kentucky, Mississippi, and Tennessee) and West South Central (Arkansas, Louisiana, Oklahoma, and Texas) divisions. Moreover, divisions are relatively homogeneous in terms of policy environments, culture, and other state-level contextual factors that likely impact population health outcomes for children, adolescents, and young adults.

## 2. Trends in US mortality: The impact of early and external deaths

The recent troubling rise in US mortality rates has received increasing attention from both population health scholars and the national media because it runs counter to the long-running narrative of increasing life expectancy in the United States, and perhaps more importantly, it is characterized by unexpected changes in the age patterns and causes of death typical of US mortality. For example, Case and Deaton’s (2015) widely referenced report finds increases in all-cause mortality among middle-aged (45 to 54) non-Hispanic white people in the United States between 1999 and 2013 – a shocking finding amid a decades-long trend in declining mortality (Ma et al. 2015). A more recent report marked the third consecutive year of declines in US life expectancy (Ahmad and Bastian 2018). To put this trend in perspective, the last time US life expectancy declined in consecutive years was in 1962 and 1963 (Centers for Disease Control and Prevention 2017); that two-year decline was caused by an influenza outbreak that was abated by 1964 (US Department of Health, Education, and Welfare 1966).

The recent decrease in US life expectancy has been driven almost entirely by a dramatic surge in the mortality rate for a number of key external causes of death, raising important questions and concerns about their potential impact on future life expectancy. Most notably, the increase in all-cause mortality for young and middle-aged white people in the United States observed by Case and Deaton (2015; 2017) was and continues to be driven primarily by deaths from drug overdoses, suicides, and alcohol-related liver disease – collectively referred to as ‘deaths of despair’ due to the hypothesized socioeconomic and psychosocial nature of their etiology. Subsequent work by Masters, Tilstra, and Simon (2017a; 2017b) demonstrates the limitations of this ‘despair’-based narrative, yet continues to emphasize the dramatic rise in drug-related mortality for US men and women ages 25 to 54 over past decades.

The US mortality disadvantage relative to its high-income peers has emerged over the past 50 or so years (National Research Council 2013; Cunningham, Walton, and Carter 2018; Gillespie, Trotter, and Tuljapurkar 2014). In an important analysis of the US disadvantage, Ho (2013) finds that relative to its high-income peer countries, 66% of the US male and about 40% of the US female disadvantage in life expectancy occurred at ages less than 50. The key causes of death responsible for higher US male and female mortality at ages 0 to 50 included homicide, transportation-related injuries, and other unintentional injuries (such as drug overdoses and drownings).

Although much work has examined US adult mortality, US early life mortality remains understudied, which is surprising for several reasons. To begin, children, adolescents and young adults in the 1 to 24 age group, with a population of approximately 100.8 million, make up 31.2% of the entire US population.^7^Although this age group is generally healthy, considerable evidence suggests that many of the same social disparities and patterns in health observed among older adults are already observable at these younger ages (Mehta, Lee, and Ylitalo 2013; Mulye et al. 2009; Park et al. 2014; Rogers et al. 2017). In addition, the majority of deaths in this age group are from external causes such as motor vehicle accidents, suicides, homicides, and drug overdoses, which are considered largely preventable and avoidable (Cunningham, Walton, and Carter 2018; Singh et al. 2013).

Most troubling, as the adult rates for suicides and drug overdoses have climbed in recent years, evidence suggests the possibility of an ‘echo effect’ at younger ages. For instance, national trends in child and adolescent hospitalization for opioid poisonings continue to rise (Allen et al. 2017; Gaither et al. 2016). For example, recent work by Kane and colleagues (2018) find that pediatric intensive care unit admissions stemming from opioid ingestion among youth ages 1 to 17 increased by 35% between 2004 and 2015. Furthermore, over a comparable time period (2008–2015), suicide ideation and suicide attempts among US children and youth increased (Plemmons et al. 2018). Plemmons and colleagues (2018) also document a nearly 300% increase in suicide ideation– and attempt-related medical encounters for children and adolescents 5 to 17 years of age. Given the observed increases in some external causes of death among adults, coupled with an increase in the types of health behaviors associated with these causes of death among children, adolescents, and young adults, a closer examination of mortality in early life is of clear importance.

## 3. The southern disadvantage in US mortality

In recent years, a growing body of work has documented wide (and widening) geographic disparities in health and mortality within the United States. Regardless of the geographic level of analysis (e.g., county, division, region, and state), most studies find the southeastern United States has a significant mortality disadvantage compared to the rest of the country; this disadvantage emerged and worsened over the past 40 years (Fenelon 2013).

Understanding trends and variations in state-level mortality is important because states enact policies that are directly related to critical health exposures. First, states establish their own laws and policies regarding taxes, social safety net programs, education expenditures, minimum wages, regulation of labor markets, and public health policies. Such factors shape the health and longevity of the populations within them in a variety of ways, such as moderating the association between one’s educational attainment and mortality risk (Montez, Zajacova, and Hayward 2016; Montez 2017). People living in states and regions also tend to share common histories, cultures, and practices, which contribute to the health and mortality of those populations. Of particular relevance to early life mortality are state-level policies and contexts that may affect external causes of death, such as motor vehicle fatalities and gun-related suicides, homicides, and unintentional injuries (Cummings et al. 1997; Eichelberger, Chouinard, and Jermakian 2012; Irvin et al. 2014; Lanza 2014; Miller, Hemenway, and Azrael 2007; Williams 2009).

Only a handful of studies have examined geographic disparities in mortality rates among adolescents and young adults (ages 15 to 24) by county, state, and region (Singh 2010; Singh et al. 2013). For instance, Singh (2010) finds that for the period 2005–2007, youth mortality was highest in states located in the Southeast and lowest in states located in New England. In subsequent work, Singh and colleagues (2013) find that much of this southern disadvantage is driven by sustained socioeconomic deprivation at the county level, which, combined with higher risks of death in both urban and rural settings, contributes to higher levels of mortality from homicide, suicide, unintentional injuries (primarily from motor vehicle accident deaths), and HIV/AIDS. Moreover, southern rural settings have been associated with some of the highest age-specific mortality rates in the country, contributing to a wider urban–rural mortality gap (Cossman et al. 2010; James, Cossman, and Wolf 2018), which some studies suggest may be impacted by state-level factors such as expenditures on public health (Cossman, James, and Wolf 2017; James 2014).

The studies discussed above underscore the role of state- and division-level contexts in shaping population health outcomes. However, scant work has focused on mortality in early life. Our study seeks to contribute to this body of literature by documenting temporal trends and geographic patterns in US early life mortality and by investigating which causes of death are most responsible for contemporary geographic disparities in early life mortality. In documenting these trends and geographic patterns and investigating the key causes of death behind the contemporary geographic disparities, we aim to shed light on how state-level actions and interventions may help to dramatically reduce preventable deaths in early life.

## 4. Data and methods

To answer our research questions, we combine information from US vital statistics mortality data, also known as the Multiple Cause of Death (MCD) files, with population estimates from the US Census. The MCD files cover a 50-year period ranging from 1965 to 2014. These data are made available by the National Center for Health Statistics and include information on the decedent’s age, sex, race, state of residence, and the underlying cause of death. For years 2005–2014, geographic identifiers are available only by special request; thus, we use the restricted files with this information for those years. State population (denominator) data come from US decennial censuses or intercensal estimates.^8^ Because early life mortality is a relatively rare event, the MCD files are the only data source that provides enough cases for analysis over such a long time frame.

To answer our first research question about trends in geographic variation in early life (ages 1 to 24) mortality, we document all-cause mortality rates at the census division and state levels. We calculate sex-specific age-standardized^9^ death rates for each census division and state and for each year 1965–2014. Census divisions are subregional state groupings established by the Census Bureau to reflect similar geographic, socioeconomic, topographic, and cultural areas of the country (US Census Bureau 2020). Table 1 displays the nine census divisions and the states included within them. Although the Census Bureau includes the District of Columbia in the South Atlantic division, given our focus on states, we exclude it from our analysis.

To address our second research question, we focus on the 2010–2014 data to examine the cause of death contributions (for the five leading causes of death) to contemporary geographic disparities in all-cause mortality. We calculate the percent contribution of the five leading causes of death and deaths from all other causes to disparities in all-cause mortality between the two most consistently disadvantaged divisions (East South Central and West South Central) and the other census divisions. The all-cause mortality rates for each census division are the sum of the five cause-specific rates and the rate of death from all other causes. Therefore, the percent contribution of each cause of death to the disparity in all-cause mortality between census divisions is calculated as

$$pct.\;contribution^{obs}=\frac{\left(CSMR^{i}-CSMR^{j}\right)}{\left(ACMR^{i}-ACMR^{j}\right)}\times 100,$$

where *CSMR*^*i*^ is the cause-specific mortality rate in division *i* and *CSMR*^*j*^ is the cause-specific mortality rate in the comparison division *j*. Similarly, *ACMR*^*i*^ represents the all-cause mortality rate in division *i* and *ACMR*^*j*^ is the all-cause mortality rate in the comparison division *j*.

We split this portion of the analysis into two age groups: children (1 to 14) and adolescents and young adults (15 to 24) to account for the considerable variation in levels of mortality and causes of death between the two age groups. Given the narrow age and year range for this part of the analysis, we treat these as age-specific death rates. Although we recognize it is customary – and typically necessary – to present age-standardized death rates when comparing mortality across geographic entities, we present the age-specific all-cause and cause-specific rates. As a sensitivity check to ensure that variations in age structure across states and divisions did not skew these results, we also calculated age-standardized rates; those results (available upon request) were nearly identical to the age-specific rates. We focus our presentation of the results on the 15 to 24 age group given that the majority of deaths occur during these ages. For the interested reader, we include the results for the 1 to 14 age group in the Appendix.

Causes of death are coded using the 10^th^ Revision of the International Classification of Disease (ICD-10; World Health Organization 2007). We combine individual ICD-10 codes, grouping them into the top five leading causes of death for each age group and calculate cause- and sex-specific rates of death at the state and census division levels. To focus on the specific leading causes of death in early life, we separate unique causes that are often collapsed into a single category.^10^ For instance, motor vehicle accidents (MVAs), drownings, fires and explosions, and drug poisonings are often coded together as ‘unintentional injuries.’ Instead of collapsing these causes of death into a single category, we consider them distinct causes because MVAs were by far the leading accidental cause of death in the 1 to 24 age group, and the remaining three constituted the leading causes of nontransport accidental deaths. Similarly, homicide by firearm is often coded under the broad ‘homicide’ category, but we code it separately because 78.2% of all homicide victims in our data were killed with a firearm.^11^ Although we recognize that a significant number of suicides are also committed with a firearm, we code all suicides under one category.

## 5. Results

**Figure 1:**
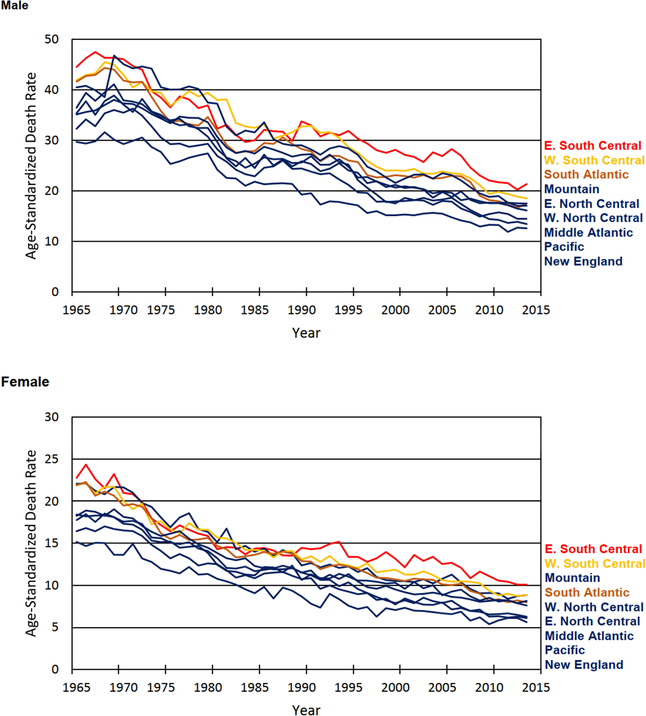
Trends in all-cause mortality, ages 1 to 24, by sex and US census division, 1965–2014 *Source*: Multiple Cause of Death files, 1965–2014. *Notes*: Age-standardized death rates (ASDRs) are presented per 100,000. The red, yellow, and brown lines represent the three southern census divisions (E. South Central, W. South Central, and South Atlantic), respectively. Census divisions are listed to the right of the graph in descending order according to their level of mortality in 2014.

Figure 1 depicts trends in age-standardized all-cause early life mortality by census division and sex between 1965 and 2014. The census divisions are labeled to the right of the graphs according to their mortality levels (highest to lowest) in 2014. All-cause mortality rates for ages 1 to 24 declined dramatically across the board during the study period; the southern divisions were no exception. For males and females, the sharpest declines in all-cause mortality occurred during the first half of the study period, between 1965 and 1989, with more modest declines between 1990 and 2014. There were fluctuations across the time period. For example, all-cause mortality increased in the early 1990s and mid-2000s for the East South Central division. Considering the West South Central division, we see that among males, all-cause mortality increased during the late 1980s and early 1990s, dropped during the late 1990s, and was somewhat stable during the early 2000s before dropping again during the past ten years of the study period. Nevertheless, the East South Central remained the most disadvantaged and the West South Central remained the second most disadvantaged census division for both males and females as of 2014.^12^The South Atlantic division fared slightly better. Even though the South Atlantic division was the third most disadvantaged division among males as of 2014, the differences in all-cause mortality between the South Atlantic and the Mountain division were slim. Moreover, among females, all-cause mortality rates were slightly higher in the Mountain division compared to the South Atlantic as of 2014. The all-cause early life mortality rates in the South Atlantic division remained substantially higher over the entire 50-year study period compared to the most advantaged divisions: New England, Pacific, and Middle Atlantic.

Overall, the results in Figure 1 reveal a consistent southern disadvantage in early life mortality from 1965 to 2014. This finding differs from the pattern of southern divergence others have documented for adults. Among adults, mortality rates were more similar across geographic contexts in the 1960s, with a southern disadvantage emerging over time as other regions improved more quickly (Fenelon 2013). Similar to adults, the results in Figure 1 show a contemporary mortality disadvantage for children, adolescents, and young adults living in the South, particularly for those living in states located in the East South Central and West South Central census divisions.

**Figure 2:**
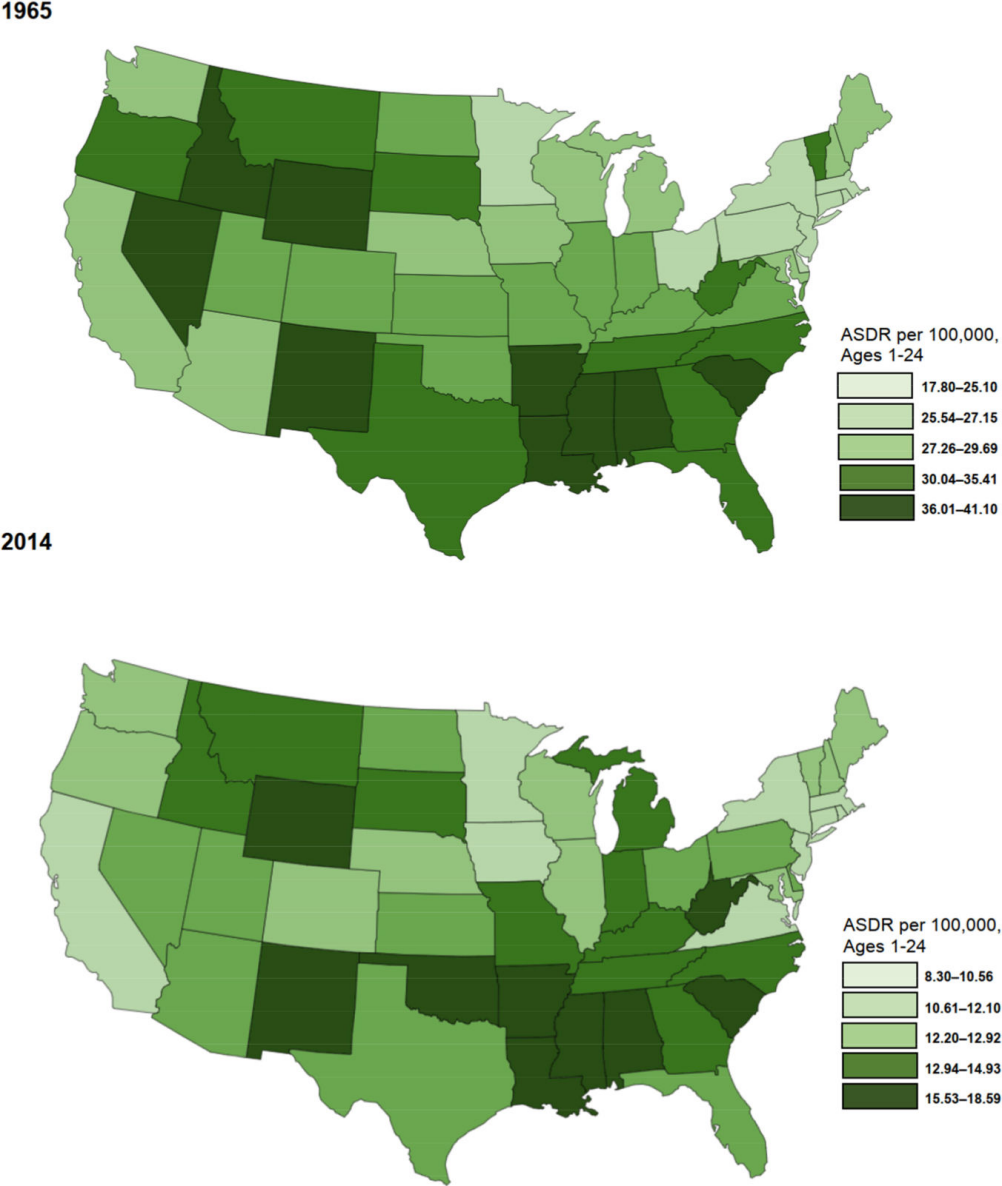
Age-standardized mortality by US state, ages 1 to 24, 1965 and 2014 *Source*: Multiple Cause of Death files, 1965 and 2014. *Notes*: States are classified into quintiles. Darker shades represent higher death rates. Death rates are standardized to the 2000 US age structure.

Figure 2 further illustrates this point and shows which states exhibited the highest rates of early life mortality in 1965 and 2014, respectively, with the highest rates of mortality (i.e., the fifth quintile) represented by darker shading. A southern mortality disadvantage was already quite clear in 1965: Arkansas, Louisiana, Mississippi, Alabama, and South Carolina comprised half of the highest quintile for early life mortality in the country at that time; another six southern states (West Virginia, Tennessee, North Carolina, Georgia, Florida, and Texas) were in the second highest quintile of states. At the same time, four of the highest mortality states in 1965 were found in the Mountain division (New Mexico, Nevada, Wyoming, and Idaho).^13^ By 2014, most states in the Mountain division had improved their relative standing. For instance, Idaho improved modestly from the highest quintile in 1965 to the second highest by 2014. Colorado moved from the middle quintile in 1965 to the lowest quintile in 2014. In an even more dramatic improvement, Nevada moved from the highest quintile (and the highest age-standardized death rate in the nation) in 1965 to the middle quintile in 2014, while only two Mountain division states – Wyoming and New Mexico – remained in the highest quintile. In contrast, Figure 2 shows that most states in the South did not improve their relative standing over the 50 years, although there are exceptions. For example, Virginia improved from the middle quintile in 1965 to the lowest mortality quintile in 2014. And both Florida and Texas improved from the second highest mortality quintile in 1965 to the middle quintile in 2014. Future research could examine the reasons behind the relative improvements in early life mortality in those three southern states over the past 50 years.

In 2014, seven of the ten states with the highest early life mortality rates were concentrated in the South (Arkansas, Louisiana, Mississippi, Alabama, South Carolina, Oklahoma, and West Virginia). Indeed, between 1965 and 2014, both Oklahoma and West Virginia moved into the highest mortality quintile. Of the complete set of 16 southern states in 2014, Virginia is the only state with an early life mortality rate in the most favorable quintile; Maryland is in the second most favorable quintile; and Delaware, Florida, and Texas are in the middle quintile. The remaining southern states comprised 11 of the 20 highest early life mortality states in the country in 2014 – which is the same number of southern states comprising the highest two quintiles in 1965.

Figures 1 and 2 clearly show that all-cause mortality rates in early life declined substantially between 1965 and 2014 throughout the United States. In 2014, as in 1965, the South continues to lag behind the rest of the nation. To improve our understanding of the contemporary southern disadvantage in early life mortality, we turn to the second question on the leading causes of death in 2010–2014.

Table 2 shows the number and percent of deaths for the five leading causes of death by age group for the period 2010–2014. With the exception of malignant neoplasms of the brain among children aged 1 to 14, the five leading causes of death for both age groups are from external causes. Not surprisingly, MVAs are the leading cause of death for both age groups, comprising 13% of deaths among children and 23.6% of deaths among adolescents and young adults. The causes of death are more varied among children than among adolescents and young adults. The five leading causes of death constitute only 30.4% of all deaths among children but 65.8% of all deaths among adolescents and young adults. Moreover, 75.5% of all deaths in the 1 to 24 age group during the period occurred among adolescents and young adults (ages 15 to 24). For this reason, we focus the remainder of our analysis on these older ages.

Table 3 displays the rates of death by cause, sex, and census division for the period 2010–2014. Similar to the patterns described above, Table 3 shows that among adolescents and young adults, all-cause and cause-specific mortality are particularly high in the East South Central and West South Central divisions for both males and females. The causes of death most responsible for the heightened mortality in those two divisions are MVAs and, to a lesser extent, homicide by firearm and drownings. Although deaths from MVAs are also particularly high in the Mountain and West North Central divisions, the disparities in deaths from MVAs in the southern divisions compared to the other divisions are stark. For example, the rate of death from MVAs in the East South Central is 40.8 per 100,000, almost twice that of New England (20.6 per 100,000; see Table 3). Drug poisonings, on the other hand, do not display particularly strong division differences for either males or females. Notably, suicide mortality is much higher in the Mountain division compared with any other division for both males and females in this age group. In addition, among males, the rate of death from homicide by firearm is highest in the East North Central division (24.7 per 100,000) and comparable to the southern divisions among females. However, analysis at the census division level, such as that presented in Table 3, may mask further state-level variations in cause-specific early life mortality. Given that the rates of death among adolescents and young adults are substantially higher among males – as we would expect – we narrow the remainder of our analysis to males. The results for females aged 15 to 24 are included in the Appendix.

**Figure 3:**
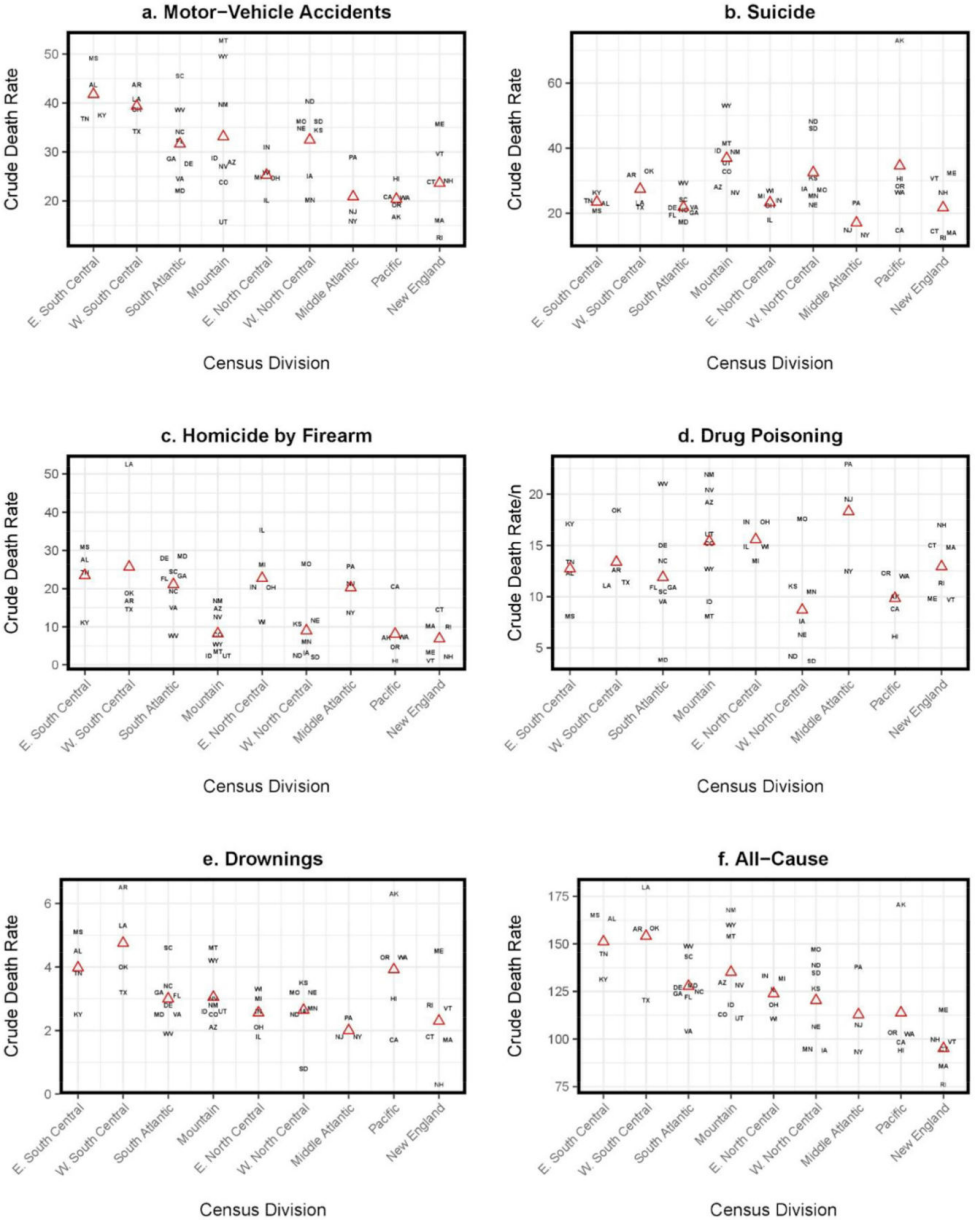
Cause-specific rates of death by state and census division for US males, ages 15 to 24, 2010–2014 *Source*: Multiple Cause of Death files, restricted use, 2010–2014. *Notes*: Cause-specific rates of death are expressed per 100,000. States are represented by their two-letter postal abbreviation and appear over their respective census division listed across the x-axis. The red triangles represent the mean cause-specific rate of death among states in the census division.

Figure 3 displays the cause-specific rates of death by state and census division among males aged 15 to 24 for the period 2010–2014. In each panel, states are grouped above their respective census division labeled across the x-axis. The red triangles represent the mean cause-specific rate of death among states in the census division. States in the East South Central and West South Central divisions, and to a lesser extent, the South Atlantic, tend to have higher rates of death from MVAs, homicides by firearm, and drownings compared to other states. For instance, the top left panel in Figure 3 shows that adolescent and young adult males in every state in the East South Central and West South Central divisions experienced a rate of death from MVAs exceeding the average MVA mortality rate for young men in very other census division. Mississippi had the highest rate of death from MVAs among southern states and the third highest overall, with a rate of death of 49.1 per 100,000. Indeed, very few individual nonsouthern states exceed the average of approximately 40 per 100,000 deaths observed in the East South Central and West South Central divisions (e.g., Montana, Wyoming, New Mexico, and North Dakota). Moreover, four of the eight states in the South Atlantic division (South Carolina, West Virginia, North Carolina, and Florida) had a rate of deaths from MVAs above 30 per 100,000 – on par with the average for the Mountain division and the West North Central division – but higher than most other states. Furthermore, there is more variation between states in all other divisions compared to the East South Central and West South Central divisions. For instance, in the Mountain division, Montana and Wyoming had the highest rates of death from MVAs in the nation, but the remaining states in that division had relatively low rates of death from MVAs, with Utah having among the lowest in the country. The higher rates of mortality due to MVAs combined with less variability between states for MVA mortality within the East South Central and West South Central census divisions provides striking evidence of one potential cause for a southern disadvantage in early life mortality.

We also see a distinct disadvantage among southern states with respect to homicide by firearm – shown in panel c of Figure 3. Although there is more variation among southern states in rates of death from homicide by firearm compared to MVAs, from 2010 to 2014, 11 of the 20 states with the highest rates of death from homicide by firearm were concentrated in the South. Most striking, in the West South Central division, adolescent and young adult males in Louisiana experienced a rate of death from homicide by firearm of 52.5 per 100,000 – by far the highest in the nation. Other highlights from Figure 3 include that the high rates of death from homicide by firearm in the East North Central division are driven largely by Illinois and, to a lesser extent, Michigan. We discuss the potential roles of race/ethnicity and urban–rural contexts in the Discussion section. The highest rates of death from suicide are concentrated among states in the Mountain division, although North Dakota, South Dakota, and Alaska, in the West North Central and Pacific divisions, respectively, also had high rates of death from suicide. This pattern mirrors the “suicide belt” researchers have documented for adults (Smith and Kawachi 2014). Despite the high rates of death from suicide in the Mountain division states, and lack of a clear geographic patterning in rates of death from drug poisoning, the data presented in Table 3 and Figure 3 shows a substantial disparity in all-cause mortality among adolescent and young adult males in most states concentrated in the East South Central and West South Central divisions. Due to this persistent patterning of results, we conclude our analysis by assessing the contribution of each leading cause of death to observed differences in all-cause mortality between these two southern divisions and all other census divisions for the period 2010–2014.

Table 4 clearly shows that the southern disadvantage in all-cause mortality among adolescent and young adult males is fueled primarily by disparities in rates of death caused by MVAs and homicide by firearm. For example, the first column of Table 4 shows that the difference in the all-cause mortality rate between adolescent and young adult males in the East South Central versus New England divisions was 58.4 per 100,000. MVAs (34.6%) and homicide by firearm (23.4%), taken from columns 2 and 4, respectively, account for 58% of that disparity. Similarly, 52.9% of the disparity in all-cause mortality rates between the East South Central and Pacific divisions was attributable to deaths caused by MVAs (40.4%) and homicide by firearm (12.5%), respectively. Even in comparison to the East North Central – a division with slightly higher rates of death due to homicide by firearm (see Table 3) – Table 4 reveals a substantial elevation in all-cause mortality for the East South Central division (25.5 per 100,000), 64% of which was attributable to deaths from MVAs. Similarly, Table 4 shows higher all-cause mortality for the East South Central compared to the Mountain division (22.3 per 100,000) and the West North Central Division (31.2 per 100,000), despite both of those divisions’ higher rates of death due to suicide.

The results in Table 4 comparing the West South Central to the other census divisions highlight how the early life mortality penalty is not as severe as it is for the East South Central division. Nevertheless, Table 4 shows some similar patterns, with a few notable differences. For instance, the difference in all-cause mortality among adolescent and young adult males in the West South Central division compared to the New England division is 42.6 deaths per 100,000; MVAs (36.5%) and homicide by firearm (23.9%) account for 60.4% of that disparity. Perhaps most striking is the comparison between the West South Central and the West North Central, in which a staggering 85.1% of the difference in all-cause mortality (15.5 per 100,000) is attributed to MVAs (37.5%) and homicide by firearm (47.6%). Finally, the difference in all-cause mortality between the West South Central and the Mountain divisions is 6.5 deaths per 100,000. Although the majority of this disparity is due to MVAs and homicide by firearm, drownings account for 17.5% of the West South Central disadvantage. In general, the results in Table 4 indicate that drownings play a larger role in explaining the West South Central division’s relative early mortality disadvantage than that of the East South Central. Nevertheless, the results from Table 4 clearly demonstrate that the contemporary southern disadvantage in early life mortality is mostly attributable to disparities in deaths due to MVAs, homicide by firearm, and suicide.

## 6. Discussion

Our findings show that the temporal trends in US early life mortality are different from those that have been documented for adults, even though the contemporary geographic patterning is similar. Unlike adults, the mortality disadvantage for southern children, adolescents, and young adults did not emerge in the 1970s or 1980s; rather, the trends in our analysis show evidence of excess early life mortality in the South across the entire 50-year period, 1965–2014. Our findings also demonstrate that mortality rates among children, adolescents, and young adults in the United States declined substantially between 1965 and 2014. Although these declines were observed nationwide, similar to adults, the data show a contemporary disadvantage in all-cause mortality for young people ages 1 to 24 living in states located in the South compared to those living in other states and divisions of the country. Our results also show that seven of the ten states with the highest contemporary early life mortality rates are located in the South (Arkansas, Louisiana, Mississippi, Alabama, South Carolina, Oklahoma, and West Virginia), five of which are located in the East South Central and West South Central census divisions – the two divisions that have been, and remain, the most disadvantaged.

Our cause-specific mortality analysis shows that young people living in the South, regardless of sex, tend to experience higher rates of death from MVAs, homicide by firearm, and to a lesser extent, suicides and drownings – all of which are external and preventable causes of death. These findings are consistent with past research (e.g., Montez, Hayward, and Wolf 2017), which suggests that geography intersects with other fundamental causes and upstream determinants of health and mortality and thus contributes to the southern disadvantage, especially in early life. It is likely that there are contextual state- and local-level social, economic, and political factors that contribute to the southern early life mortality penalty, including systemic and institutionalized racism (Phelan and Link 2015). Given the well-documented mortality penalty for African American people (Williams and Sternthal 2010), coupled with the fact that 58.8% of African American children, adolescents, and young adults live in the South,^14^ a reasonable conclusion from our analysis is that high levels of early life mortality observed in the South may be attributable, at least in part, to the high concentration of African American people who are faced with the enduring legacy of Jim Crow laws. However, additional calculations (available upon request) indicate that the southern disadvantage in early life mortality is most pronounced among white people.^15^ Although a thorough examination of the role of race/ethnicity in shaping our results is beyond the scope of this study, our ongoing research is examining this topic in detail.

Another potential underlying cause of the southern disadvantage is poverty, which would be consistent with the findings from Singh and colleagues (2013) and Currie and Schwandt (2016), who find a strong association between early life mortality and geographic socioeconomic deprivation^16^ and status, respectively. Indeed, 12 of the 20 states with the highest levels of poverty are concentrated in the South, with Mississippi and Louisiana topping that list (US Census Bureau 2016). If poverty is a driving factor of the southern disadvantage in early life mortality, this may help explain the relative advantage of the South Atlantic among the southern census divisions. The South Atlantic division is more heterogeneous and includes Delaware, Maryland, and Virginia, states characterized by relatively low levels of poverty and high incomes bolstered by federal employment (Zieger 2012) and commensurate lower levels of all-cause mortality. In comparison, all eight states in the East South Central and West South Central divisions are in the top quintile for poverty. Efforts in these states to ameliorate the deleterious effects of poverty would most certainly contribute to improved population health outcomes for all ages. Unfortunately, the political will for such efforts in many of these states is often lacking. Among the most notable recent examples is the failure of most southern states to expand Medicaid coverage as part of the Patient Protection and Affordable Care Act. For instance, as of January 4, 2019, 9 of the 14 states that have not adopted Medicaid expansion were located in the South; among the eight states in the East South Central and West South Central divisions, only three (Arkansas, Louisiana, and Kentucky) have elected to do so (Kaiser Family Foundation 2018).

Our findings suggest that deaths in early life, and geographic disparities therein, could also be reduced – perhaps more expediently – with the adoption and implementation of specific policies at the state level targeted at preventing the major causes of death in this age group. Specifically, our analysis indicates that for the period 2010–2014, between 52.9% and 68.4% of the disparities in all-cause mortality between adolescent and young adult males in the East South Central and West South Central divisions and their peers in the New England, Pacific, and Middle Atlantic divisions were attributable to deaths caused by MVAs and homicide by firearm. The application of targeted efforts to prevent early life deaths from these external causes in southern states could dramatically reduce these disparities. For instance, analyzing traffic safety data spanning the latter half of the 20^th^ century, several analyses consistently demonstrate the significance of state regulatory environments in reducing motor vehicle fatalities. Importantly, state actions can change drivers’ behaviors by mandating the use of seat belts, enacting graduated driver’s license regulations, increasing the minimum legal drinking age, and instituting lower maximum speed limits (Calkins and Zlatoper 2001; Houston, Richardson Jr, and Neeley 1995; 1996; Houston 1999; Williams 2009). These findings are substantiated by multiple reports from the Centers for Disease Control and Prevention documenting the significant state- and regional-level variation in motor vehicle behavior and fatalities across the United States. For instance, approximately one in five child passenger deaths in the United States involves an alcohol-impaired driver, with the highest rates in South Dakota, New Mexico, and Mississippi (Quinlan, Shults, and Rudd 2014). In addition, despite a considerable body of research documenting the importance of booster seat laws in reducing child injuries and fatalities (Eichelberger, Chouinard, and Jermakian 2012; Mannix et al. 2012), nearly one in three child motor vehicle deaths occur among unrestrained youth (Sauber-Schatz, West, and Bergen 2014). Moreover, there are considerable temporal, interstate differences in the requirements of child passenger safety laws (Bae et al. 2014).

State contexts are also important predictors of geographic variation in gun-related mortality. Despite the lack of research funding and publications on gun violence relative to its impact on population health (Macinko and Silver 2012), there is general consensus that broader availability of firearms is strongly associated with higher rates of death due to homicide, suicide, and unintentional firearm deaths (Kalesan et al. 2016; Miller, Hemenway, and Azrael 2007; Price, Thompson, and Dake 2004; Santaella-Tenorio et al. 2016; Siegel, Ross, and King 2013; Siegel, Ross, and King 2014). Much of the work in this area is specifically concerned with the role of state-specific legislative actions in helping to either tighten or loosen gun restriction criteria and the influence of these critical decisions on mortality rates. A number of studies note how state background checks for gun purchase and restrictions on ownership are strongly associated with reductions in firearm-related homicide and suicide deaths (Andrés and Hempstead 2011; Sen and Panjamapirom 2012; Sumner, Layde, and Guse 2008), as they may impose additional obstacles preventing unauthorized individuals from obtaining firearms in the secondary market (Ruddell and Mays 2005), or eliminate a possible means of suicide among at-risk individuals (Brent and Bridge 2003; Shenassa et al. 2004).^17^ Additionally, state regulation of firearm dealers is crucial, as homicide rates are lower in states that require the licensing and regular inspection of firearms dealers (Irvin et al. 2014). For example, Webster and colleagues (2014) find that firearm homicide rates in Missouri increased by 23% following the repeal of its permit-to-purchase handgun law, which mandated compliance on the part of both purchaser and dealer in guaranteeing the legitimacy of a firearm purchase. Overall, both the greater quantity of firearms-related legislation and higher quality of gun control policies are consistently associated with lower rates of firearm-related mortality, thus helping to account for the considerable variation in firearm-related mortality rates across states (Fleegler et al. 2013; Kalesan et al. 2016; Lanza 2014; Santaella-Tenorio et al. 2016; Simonetti et al. 2015).

Though research on early life firearm-related mortality is even sparser than that among adults, the majority of studies emphasize the significance of firearm laws and regulations in reducing gun-related mortality among young people. For instance, states that hold gun owners responsible for keeping firearms secure from children (i.e., Child Access Prevention [CAP] laws) experience a greater decline in the rate of unintentional firearm deaths for children (Cummings et al. 1997; Hepburn et al. 2006; Webster and Starnes 2000). These laws are particularly effective in reducing youth suicides, which are highly preventable if and when access to guns and ammunition is restricted (Grossman et al. 2005; Webster et al. 2004). In a similar vein, while finding mixed results for the effectiveness of CAP laws, Lee and colleagues (2013) document a significant increase in firearm injuries in states that have instituted a Stand-Your-Ground law, which authorizes the use of firearms stemming from a perceived need for self-defense. Overall, studies find that more ‘lenient’ states have higher rates of unintentional and self-inflicted firearm-related injuries than their legislatively strict counterparts (Safavi et al. 2014; Tashiro et al. 2016). Given the higher firearm availability in western and southern states (Brown, Imura, and Osterman 2014; Kalesan et al. 2016), combined with the generally less restrictive gun laws in many of these states (Santaella-Tenorio et al. 2016), it is perhaps unsurprising that the limited extant research on this subject finds elevated pediatric firearm homicides narrowly concentrated among southern states (McBride 2018). Although our primary focus in this article is on the southern disadvantage in early life mortality, reducing firearm availability could also prevent many of the early life deaths attributed to suicide in the Mountain division states. Indeed, approximately 50% of suicides among children, adolescents, and young adults in the Mountain division states are committed with a firearm.^18^

Vital statistics data allowed us to thoroughly document trends and patterns in early life mortality in the United States but do not have the time-varying, state-level variables necessary to assess explanations for the patterns and trends we document. We hope that our results spur future research in this area, particularly regarding how policy contexts shape the early life mortality patterns and trends documented herein. In particular, there are a few striking patterns that merit future research. One is the improvement over time in early life mortality in Mountain division states such as Nevada, relative to other states. There are multiple potential explanations for the shift in Nevada, including demographic changes. Nevada has exhibited the largest proportional population increase since 1950 of any US state, most of which is attributed to in-migration.^19^ It may be that the shifting demographic context is consequential for behaviors, norms, or policies related to early life mortality.

In addition, our data do not have the detail to directly address concerns about urban–rural mortality gaps (Cossman et al. 2010; James 2014). Future research could apply innovative data linkages to formally model variation in urban–rural early life mortality gaps across state contexts, extending prior descriptive research (James, Cossman, and Wolf 2018). Extant research on geographic variation in adult mortality has been increasingly moving in this direction, linking large-scale high-quality survey data to diverse administrative and historical data sets to better understand how macro-level contexts and changes influence region and even state-specific health outcomes (Hayward et al. 2018; Montez, Hayward, and Wolf 2017; Montez et al. 2019), including mortality (Montez, Hayward, and Zajacova 2019). While studying early mortality represents a greater challenge for these analyses given the smaller number of deaths, we believe that demographic and social research should continue to advance in this direction. Linking cause-specific mortality at younger ages to individuals’ state-level social, demographic, and economic contexts can better identify the types of state policies and legislative decisions that most effectively impact the health and longevity of children, adolescents, and young adults.

## 7. Conclusion

This paper highlights a persistent mortality penalty for children, adolescents, and young adults living in the southern United States over the entire 50-year period of 1965 to 2014. The contemporary southern disadvantage in US early life mortality tends to be most pronounced in states located in the East South Central and West South Central census divisions. These findings indicate that the persistent southern mortality disadvantage – which is well documented for adults – has its origins early in the life course and is not simply due to adult health behavior patterns. This early life mortality southern disadvantage underscores the argument that examining early life mortality is critical to improving our understanding of population health and longevity. Finally, our findings reveal that the contemporary southern disadvantage in all-cause early life mortality is attributed primarily to key external and preventable causes of death – motor vehicle accidents and homicides by firearm. Our findings have direct policy implications and may assist decision makers in the South (and elsewhere) to focus on the causes of death that are especially harmful to young people. In this spirit, we urge policymakers in states that have particularly high rates of early life mortality to borrow ideas, such as particular policies and regulations, from those in other areas of the country that have reduced excess deaths for children, adolescents, and young adults with an eye toward reducing – or even eliminating – the southern mortality disadvantage in early life.

## Figures and Tables

**Table 1:** US states and their census divisions

Division	States included
East South Central	Alabama, Kentucky, Mississippi, Tennessee
West South Central	Arkansas, Louisiana, Oklahoma, Texas
South Atlantic	Delaware, Florida, Georgia, Maryland, North Carolina, South Carolina, Virginia, West Virginia
East North Central	Illinois, Indiana, Michigan, Ohio, Wisconsin
West North Central	Iowa, Kansas, Minnesota, Missouri, Nebraska, North Dakota, South Dakota
Middle Atlantic	New Jersey, New York, Pennsylvania
Mountain	Arizona, Colorado, Idaho, Montana, Nevada, New Mexico, Utah, Wyoming
Pacific	Alaska, California, Hawaii, Oregon, Washington
New England	Connecticut, Maine, Massachusetts, New Hampshire, Rhode Island, Vermont

*Source*: US Census Bureau (2015).

**Table 2:** Number and percent of deaths for five leading causes and all other causes by age group, United States, 2010–2014

Ages 1 to 14	No. of deaths	Percent of all deaths
Motor vehicle accidents	6,105	13.0%
Drownings	3,245	6.9%
Malignant neoplasm of the brain	2,041	4.3%
Suicides	1,695	3.6%
Fires and explosions	1,238	2.6%
All other causes	32,824	69.6%
Total	47,148	100.0%
Ages 15 to 24		
Motor vehicle accidents	34,339	23.6%
Suicides	24,251	16.7%
Homicide by firearm	18,936	13.0%
Drug poisonings	15,620	10.7%
Drownings	2,747	1.8%
All other causes	49,784	34.2%
Total	145,677	100.0%

*Source*: Multiple Cause of Death files, restricted use, 2010–2014.

*Note*: These figures may differ from official counts due to differences in coding of the underlying cause of death. The ICD-10 codes used for each cause of death are: motor vehicle accidents (V02-V04,V09.0,V09.2,V12-V14,V19.0-V19.2, V19.4-V19.6,V20-V79,V80.3-V80.5,V81.0-V81.1,V82.0-V82.1,V83.0-V83.6, V84.0-V84.4, V85.0-V85,4, V86.0-V86.3, V87.0-V87.5, V87.7-V87.8, V88.0-V88.8, V89.0,V89.2); suicides (X60-X84, Y87.0); homicide by firearm (X93-X95); malignant neoplasm of the brain (C71.0-C71.9); drug poisonings (X40-X44); drowning (W65-W74); and fires and explosions (X00-X09).

**Table 3:** Death rates by cause, sex, and census division in the United States, ages 15 to 24, 2010–2014

	MVA	Suicide	Homicide by firearm	Drug poisoning	Drowning	All other	All-cause
**Males**							
E. South Central	40.8	23.7	23.2	13.1	3.9	45.2	149.9
W. South Central	36.1	23.8	19.7	12.2	3.8	38.5	134.1
South Atlantic	31.0	20.7	21.5	10.8	3.1	36.4	123.5

Mountain	27.9	33.0	9.8	16.9	2.6	37.3	127.6
E. North Central	24.5	22.6	24.7	15.5	2.4	34.7	124.4
W. North Central	30.4	28.5	12.4	11.2	2.9	33.3	118.7
Middle Atlantic	20.3	16.7	19.0	17.2	2.0	34.8	109.9
Pacific	20.7	18.5	17.0	9.4	2.3	32.3	100.2
New England	20.6	17.4	9.5	14.1	2.0	27.9	91.5

**Females**							
E. South Central	18.8	4.5	3.1	5.0	0.4	28.8	60.6
W. South Central	15.2	5.5	2.9	4.3	0.4	23.1	51.4
South Atlantic	12.4	4.7	2.9	4.3	0.3	22.3	46.9

Mountain	12.9	9.3	2.1	6.2	0.7	21.6	52.8
E. North Central	10.9	5.5	2.9	5.7	0.3	21.8	47.1
W. North Central	14.0	6.4	2.1	3.9	0.3	19.6	46.3
Middle Atlantic	7.2	4.5	1.5	5.5	0.2	19.1	38.2
Pacific	7.9	5.0	1.7	3.1	0.2	17.3	35.3
New England	7.7	4.8	1.0	4.8	0.2	15.6	34.1

*Source*: Multiple Cause of Death files, restricted use, 2010–2014.

*Note*: Sex-age-specific rates of death are presented per 100,000 by cause in that division.

**Table 4:** Percent contribution of each leading cause of death to the central southern disadvantage in all-cause mortality among males in the United States, ages 15 to 24, 2010–2014

			Percent contribution of overall difference due to:				
E. South Central relative to:	Diff. in all-cause mortality	MVAs	Suicide	Homicide by firearm	Drug poisoning	Drownings	All other
W. South Central	15.8	29.5	−0.8	21.9	5.8	0.9	42.7
South Atlantic	26.4	36.9	11.3	6.4	8.73	3.1	33.5
Mountain	22.3	57.9	−41.6	59.8	−17.2	5.8	35.4
E. North Central	25.5	64.0	4.4	−5.8	−9.7	5.9	41.1
W. North Central	31.2	33.5	−15.4	34.6	5.9	3.3	38.2
Middle Atlantic	40.0	51.3	17.6	10.4	−10.2	4.9	26.1
Pacific	49.7	40.4	10.4	12.5	7.4	3.2	26.1
New England	58.4	34.6	10.8	23.4	−1.8	3.3	29.7

**W. South Central relative to:**							
South Atlantic	10.6	47.9	29.5	−16.6	13.2	6.25	19.8
Mountain	6.5	126.4	−140.2	151.4	−72.7	17.5	17.6
E. North Central	9.7	120.2	13.0	−50.9	−34.8	13.9	38.6
W. North Central	15.5	37.5	−30.4	47.6	6.0	5.7	33.6
Middle Atlantic	24.2	65.5	29.6	2.9	−20.7	7.5	15.2
Pacific	33.9	45.5	15.7	8.0	8.2	4.2	18.3
New England	42.6	36.5	15.2	23.9	−4.7	4.2	24.9

*Source*: Multiple Cause of Death files, restricted use, 2010–2014.

*Note*: Differences in all-cause mortality are expressed per 100,000 in the first column. The percent contributions of the overall differences due to each leading cause of death appear in the columns to the right. A negative percent contribution indicates that the East South Central and West South Central division had a rate of death lower than the comparison division due to that specific cause. A percent contribution greater than (+/−) 100 indicates that the difference in mortality between divisions due to that specific cause was greater than the difference in all-cause mortality.

**Figure A-1:**
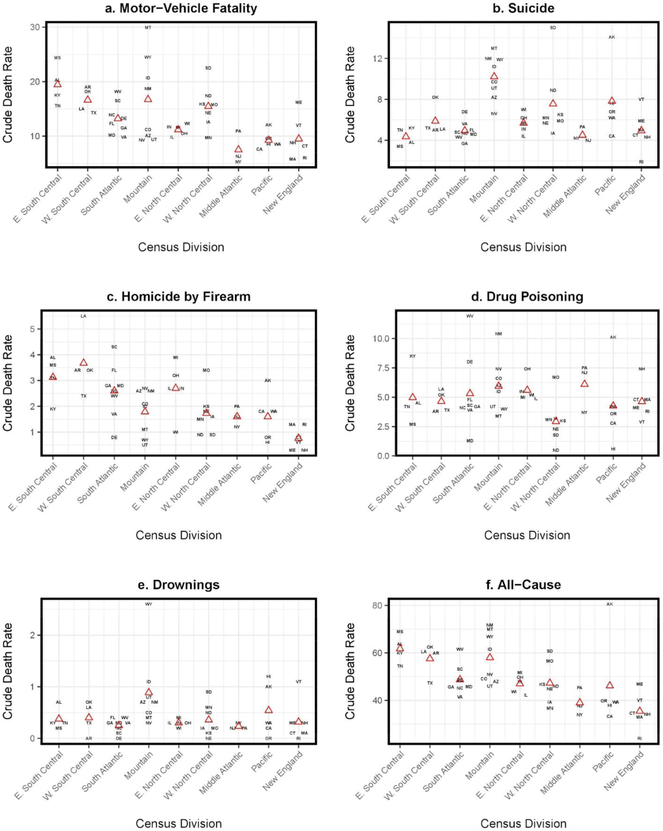
Cause-specific rates of death by state and census division for US females, ages 15 to 24, 2010–2014 *Source*: Multiple Cause of Death files, restricted use, 2010–2014 *Notes*: Cause-specific rates of death are expressed per 100,000. States are represented by their two-letter postal abbreviation and appear over their respective census division listed across the x-axis. The red triangles represent the mean cause-specific among states in the census division.

**Figure A-2:**
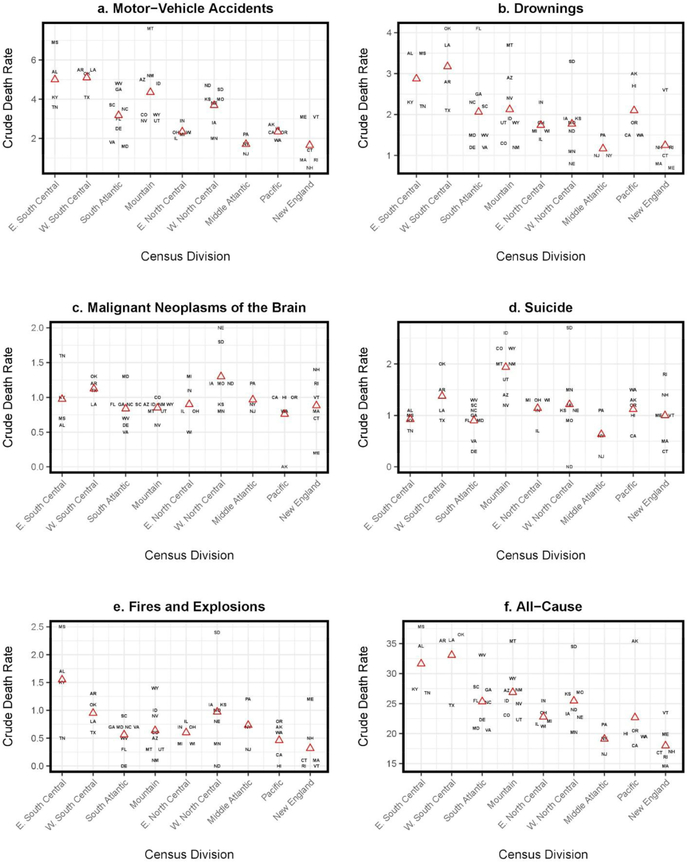
Cause-specific rates of death by state and census division for US males, ages 1 to 14, 2010–2014 *Source*: Multiple Cause of Death files, restricted use, 2010–2014 *Notes*: Cause-specific rates of death are expressed per 100,000. States are represented by their two-letter postal abbreviation and appear over their respective census division listed across the x-axis. The red triangles represent the mean cause-specific rate of death among states in the census division.

**Figure A-3:**
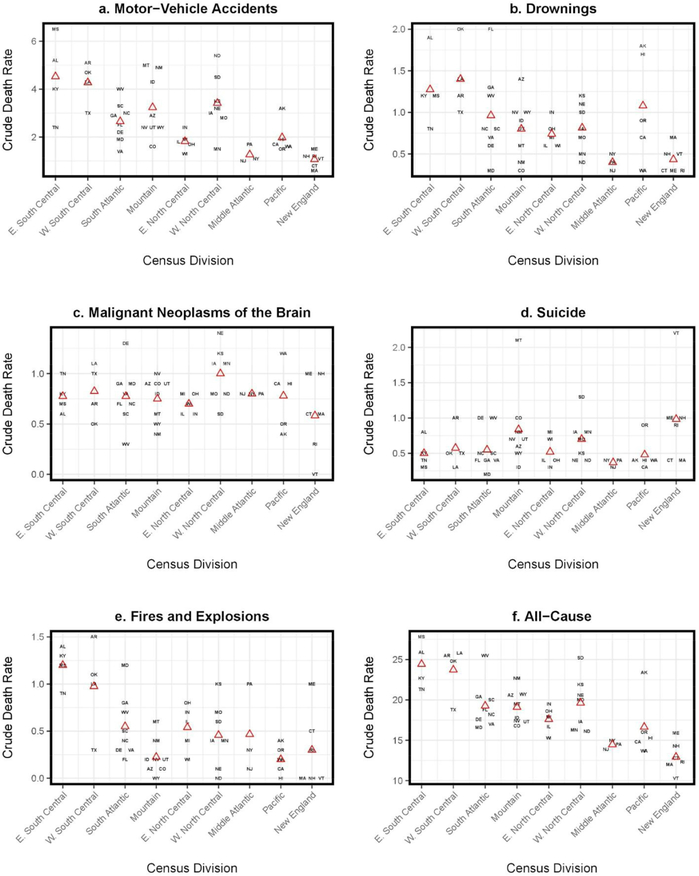
Cause-specific rates of death by state and census division for US females, ages 1 to 14, 2010–2014 *Source*: Multiple Cause of Death files, restricted use, 2010–2014 *Notes*: Cause-specific rates of death are expressed per 100,000. States are represented by their two-letter postal abbreviation and appear over their respective census division listed across the x-axis. The red triangles represent the mean cause-specific rate of death among states in the census division.

## Footnotes

6 We do not analyze infant mortality (ages 0 to 364 days) in this article for two reasons. First, the causes of infant death differ dramatically in comparison with the predominant causes of child and adolescent mortality for those ages 1 to 24 years. Most infant deaths occur during the first month of life and the determinants of infant mortality are rooted in the health and socioeconomic lives of childbearing-aged women. Given such a difference in the causes of infant death from those occurring to children and youth – which are largely due to unintentional injuries, homicides, and suicides – including them in the same analysis would be overly complex, and we would need to focus on some different issues than we do in the current article. Second, linked birth/infant death files are the most effective data to examine infant mortality in the United States. Including analyses from that data set in this particular article would lead to far too much complexity for one research manuscript.

7 Authors’ calculation of 2016 American Community Survey data.

8 State population estimates from 1969 to 2014 were made available by the National Bureau of Economic Research. For the years 1965 to 1968, we use the 1960 and 1970 decennial US Census state population estimates by sex and age, and interpolate those estimates for the four years spanning from 1965–1968, assuming a constant rate of growth.

9 All rates are standardized to the 2000 US age structure. We do this by first calculating the age- and sex-specific rate of death in each year, for each state, for the age groups 1 to 4, 5 to 9, 10 to 14, 15 to 19, and 20 to 24. We then multiply the age- and sex-specific rates by the proportion of that respective group to the overall 2000 US population. The sum of those adjusted age-specific rates is our age-standardized mortality rate for the 1 to 24 age group.

10 The specific ICD-10 codes used for each cause are listed in the notes at the bottom of Table 2.

11 Authors’ calculations of Multiple Cause of Death files, 2010–2014.

12 Note that among females, the age-standardized all-cause mortality rates were the same for the West South Central and Mountain divisions in 2014.

13 Although not shown in Figure 2, Alaska, in the Pacific census division, had a consistently high mortality rate throughout the study period, largely due to high rates of mortality due to unintentional injuries across the whole study period.

14 Authors’ calculations of 2016 American Community Survey data.

15 These data show a different contemporary geographic patterning in early life mortality among African American people. In particular, our calculations of 2014 death certificate data indicate that states with the highest all-cause mortality rates for African American adolescents and young adults are not as highly concentrated in the South but are more dispersed across states in the Northeast and Midwest.

16 The percentage of families below the poverty line was one of twenty-two variables Singh and colleagues used to measure socioeconomic deprivation at the county level.

17 Some argue that geographic variation in US gun culture shapes individual preferences for using firearms in committing suicide. Brown, Imura, and Osterman (2014) and Felson and Pare (2010) contend that ‘honor cultures’ promoting aggression toward oneself and others define the majority of western and southern states, and thus help account for the greater proportion of firearm-related suicides and homicides in these parts of the country.

18 Authors’ calculations of 2010–2014 Multiple Cause of Death files.

19 Authors’ calculations of US Census data.

## Appendix

**Table A-1:** Death rates by cause, sex, and census division in the United States, ages 1 to 14, 2010–2014

	MVA	Drowning	Malig. neop. of brain	Suicide	Fire	All Other	Ali-Cause
	
	Rate	Rate	Rate	Rate	Rate	Rate	Rate
**Males**							
E. South Central	4.7	2.8	1.1	0.9	1.4	20.0	30.8
W. South Central	4.5	2.5	1.1	1.1	0.7	18.0	27.8
South Atlantic	3.2	2.6	0.9	0.9	0.6	16.9	25.0

Mountain	4.1	2.1	0.9	1.8	0.5	16.2	25.6
E. North Central	2.3	1.7	0.9	1.1	0.6	16.1	22.7
W. North Central	3.3	1.6	1.2	1.2	0.9	16.2	24.4
Middle Atlantic	1.7	1.1	1.0	0.6	0.7	14.1	19.3
Pacific	2.2	1.6	1.0	0.7	0.3	12.9	18.7
New England	1.3	1.0	0.8	0.7	0.2	12.3	16.3

**Females**							
E. South Central	4.2	1.2	0.8	0.5	1.1	16.0	23.9
W. South Central	3.5	1.2	0.9	0.5	0.5	14.0	20.7
South Atlantic	2.5	1.2	0.8	0.4	0.5	13.4	18.8

Mountain	2.8	0.8	0.8	0.8	0.2	13.4	18.8
E. North Central	1.8	0.7	0.7	0.5	0.6	13.3	17.6
W. North Central	2.8	0.8	1.0	0.7	0.5	13.3	19.1
Middle Atlantic	1.3	0.4	0.8	0.4	0.5	11.2	14.6
Pacific	1.7	0.7	0.9	0.4	0.2	11.0	14.9
New England	0.8	0.5	0.6	0.6	0.3	9.8	12.7

*Source*: Multiple Cause of Death files, restricted use, 2010–2014.

*Note*: Age-specific rates of death are presented per 100,000 by cause in that division, 2010–2014.
